# How Primary Healthcare Sector is Organized at the Territorial Level in France? A Typology of Territorial Structuring

**DOI:** 10.34172/ijhpm.2024.8231

**Published:** 2024-06-11

**Authors:** Sylvain Gautier, Loïc Josseran

**Affiliations:** ^1^Research Center in Epidemiology and Population Health, Primary Care and Prevention Team, Inserm U1018, Université Paris-Saclay, UVSQ, Villejuif, France.; ^2^Department of Hospital Epidemiology and Public Health, Raymond Poincaré Hospital, GHU Université Paris-Saclay, AP-HP, Garches, France.

**Keywords:** Territorial Structuring, Primary Care, Geographical Typology, Healthcare Systems, Public Health

## Abstract

**Background:** Most the Organization for Economic Co-operation and Development (OECD) countries are currently facing the challenges of the health transition, the aging of their populations and the increase in chronic diseases. Effective and comprehensive primary healthcare (PHC) services are considered essential for establishing an equitable, and cost-effective healthcare system. Developing care coordination and, on a broader scale, care integration, is a guarantee of quality healthcare delivery. The development of healthcare systems at the meso-level supports this ambition and results in a process of territorial structuring of PHC. In France, the Health Territorial and Professional Communities (HTPC) constitute meso-level organizations in which healthcare professionals (HCPs) from the same territory gather. We conducted a study to determine, in a qualitative step, the key elements of the territorial structuring of PHC in France and, then, to develop, in a quantitative step, a typology of this structuring.

**Methods:** A sequential-exploratory mixed-method study with a qualitative step using a multiple case approach and a quantitative step as a hierarchical clustering on principal components (HCPC) from a multiple correspondence analysis (MCA).

**Results:** A total of 7 territories were qualitatively explored. Territorial structuring appears to depend on: past collaborations at the micro-level, meso-level coordination among HCPs and multiprofessional structures, diversity of independent professionals, demographic dynamics attracting young professionals, and public health investment through local health contracts (LHCs). The typology identifies 4 clusters of mainland French territories based on their level of structuring: under or unstructured (38.6%), with potential for structuring (34.7%), in the way for structuring (25.3%) and already structured territories (1.4%).

**Conclusion:** Interest in territorial structuring aligns with challenges in meso-level healthcare organization and the need for integrated care. Typologies of territorial structuring should be used to understand its impact on access, care quality, and medical resources.

## Background

**Key Messages**

**Implications for policy makers**

- In many of the Organization for Economic Co-operation and Development (OECD) countries, there is a need for integrated and comprehensive primary healthcare (PHC) services. This involves expanding provider organizations and collaboration between professionals in a population-based and territorial approach.
- The transition from a patient-centered micro-level model to a more integrated meso-level model relies on a process of territorial structuring of PHC.
- The developed geographical typology provides a comprehensive understanding of the degree of territorial structuring of PHC, revealing distinct categories of territories based on their level of structuring.
- The study highlights disparities in the territorial structuring, with major rural areas structured or in the way for structuring, while immediate outskirts of cities and peri-urban areas are less structured.
- The typology emphasizes the intersection and hybridization of public health and PHC functions within a population-centered approach.

**Implications for the public**

We provide a detailed characterization of the territorial structuring of primary healthcare (PHC) in France. It involves the organization of PHC actors at the territorial level to address population health issues including uneven access to healthcare in a context of decline in medical demographics. The results revealed varied territorial configurations and dynamics, with significant disparities of territorial structuring between regions. The typology could be valuable in epidemiological studies, impact assessments, and intervention studies: epidemiologists can utilize the framework to investigate the impact of PHC organization on population health, and researchers and policymakers can leverage it to assess the effectiveness of healthcare policies and interventions, tailoring strategies to address specific challenges within each type of territory. Moreover, it emphasized the role of public health in supporting the process of territorial structuring of PHC, aiming to address population-level health issues.

Most the Organization for Economic Co-operation and Development (OECD) countries are currently facing the challenges of the health transition,^1^ the aging of their populations and the increase in chronic diseases.^2^ These challenges modify the demand for healthcare services. Aging populations bring about complex healthcare needs, requiring more attention and tailored care, increasing needs from multiple healthcare professionals (HCPs). The growing prevalence of chronic diseases demands ongoing management and integrated healthcare solutions.^3^ That implies to articulate the health and social dimensions and to increase the number of HCPs involved, the accessibility and proximity of healthcare services.

 Effective and comprehensive primary healthcare (PHC) services are considered essential for establishing an equitable, and cost-effective healthcare system.^4^ Studies suggest that the strength of PHC improves when provider organizations are expanded to cover population groups in addition to a patient-based approach (micro-level traditional model).^5,6^ This expansion involves developing PHC at the meso-level of the healthcare system by enhancing coordination and integration, both organizationally and professionally, as proposed by Valentijn in his model of integrated care.^7^ That implies different healthcare professions, providers, and patient representatives to collaborate more closely in multidisciplinary teams within the community involving general practitioners (GPs), nurses, pharmacists, physiotherapists, dental professionals, social care services, third-sector providers (associational sector), and others. By working together, they can coordinate and support each other’s activities, delivering care and healthcare services to the same population within a specific geographic area, with a population and territorial responsibility and a population-based approach. Developing care coordination and, on a broader scale, care integration, is a guarantee of quality healthcare delivery.^8-10^ The development of healthcare systems at the meso-level, understood as an intermediate scale between the macro and micro levels, is particularly evident within the PHC sector.

 While the organization of PHC varies from one healthcare system to another,^11^ the transition from a patient-centered micro-level model to a more integrated meso-level model or the addition of this meso-level to the existing micro-level organization is observed in most countries: the “health areas” in Spain,^12^ the “local health authorities” in Italy,^13^ the “primary care networks” in Alberta (Canada),^14^ the “clinical commissioning groups” in the United Kingdom,^15^ and the “primary care clusters” in Wales.^16^ Similarly, in the United States, the federal agency Centers for Medicare and Medicaid Services has actively supported the implementation of local programs aimed at better integrating healthcare services, as well as medical-social services, like the “Accountable Care Organizations.”^17^

 This process, which can be referred to as “territorial structuring of PHC,” is considered as the set of actions and measures, differently accompanied by regional public authorities, aimed at organizing and strengthening the coordination of PHC services at the scale of a specific territory, including the establishment of local collaborations and partnerships among HCPs, healthcare facilities, and other sectors.^18^ Although the determinants (drivers and barriers) of care coordination and integration resulting from this territorial structuring of PHC are extensively documented in the literature,^19,20^ the process by which integration is implemented, particularly in its territorial dimension, has been little studied so far.^21^ Understanding this process also requires considering the characteristics of the healthcare system in which it is observed, particularly PHC system.

 France’s healthcare system features ambulatory medicine based on private practice principles (independent practice, setting the consultation fee, freedom to choose the place of practice), funded by public sources: GPs primarily receive fee-for-service remuneration, largely covered by mandatory public health insurance.^22^ Indeed, there is a system of convention between physicians and Health Insurance, which involves them signing agreements to determine the rates of medical procedures and the conditions for reimbursement by health insurance. Over the past two decades, the French government has sought to regulate and streamline the traditionally unorganized PHC sector.^23^ This has led to the introduction of new organizational principles, including the “referring physician,” recognition of general practice as a full medical specialty, and the establishment of PHC teams. The roles of various professionals have been clarified and expanded, such as advanced practice nurses. Remuneration models now incorporate some capitation or profit-sharing tied to public health objectives. For instance, the Contract for Improvement of Individual Practice (*contrat d’amélioration des pratiques individuelles*), launched in 2008, introduced capitation-based remuneration for GPs. Expanded in 2011, it became Remuneration based on Public Health Objectives (*rémunération sur objectifs de santé publique*), applicable to GPs and certain specialists, featuring 29 clinical indicators for adult patient care. This approach rewards GPs with additional payments based on indicator achievement, assessed through quality indicators.^24^

 Since 2016, PHC professionals in France have been organizing themselves at the meso-level through the creation of the Health Territorial and Professional Communities (HTPC) (*communautés professionnelles territoriales de santé*), which are similar to the primary care clusters in Wales (Supplementary file 1). The HTPC clearly distinguish from healthcare homes (HHs), which are typically stakeholders within HTPCs: HHs are involved in a form of close clinical coordination centered on the patient and the professionals involved in their care, whereas HTPCs are engaged in territorial coordination, which also involves integration with the social and medico-social sectors.^25^

 The HTPCs bring together professionals from the same territory who wish to organize themselves—at their initiative—and coordinate to improve patient care with a focus on continuity and quality of care.^26^ The extent of the territory is left to the discretion of the professionals involved in the HTPC. However, there can only be one HTPC per territory. HTPC territories generally cover multiple municipalities and can vary in size depending on the number of inhabitants they cover: from less than 40 000 inhabitants to over 175 000 inhabitants.^27^ The main missions of the HTPCs are to improve access to care, organize multi-professional care pathways around the patient, develop territorial prevention actions, and contribute to providing a response in the event of a health crisis.^25^ In return for their involvement in a HTPC, HCPs may receive a fixed payment known as a coordinated care fee (similar to when they practice in a HH or a healthcare center – HC) from the health insurance.

 At the same time, the hospital sector has been marked by the emergence of 136 territorial hospital groups (*groupements hospitaliers de territoire*), new modes of cooperation between public hospitals on a territorial scale (which is larger than that of the HTPC). Cooperation revolves around a shared medical project aimed at enabling hospitals to provide better care at lower cost, in a population-based perspective.

 The investment at the territorial level (from both the primary care sector and the hospital sector) responds to a broader trend of decentralization within the French healthcare system, materialized in 2009 with the creation of regional health agencies (*agences régionales de santé*).^28^ These structures are responsible for regional management of the healthcare system and regulation of regional healthcare provision. However, within the same region, the territorial organization of PHC can vary significantly. Whether or not they have a HTPC (as they are not present everywhere), some territories may have local health contracts (LHCs) enhancing the coordination of healthcare stakeholders and developing public health initiatives.^29^ The LHC is a tool jointly supported by the regional health agency and a territorial authority (usually one or more municipalities) to reduce territorial and social health inequalities and implement solutions for local healthcare provision.^30^ The LHC aims to facilitate care and health pathways through greater integration: with health prevention actions, improved organization of care, and medical-social support.

 All these developments, in a complex dynamic of health territorialization,^28,31^ reflect an ongoing process of territorial structuring at varying degrees depending on the territories involved. The objective of this study is to understand the key elements of the territorial structuring of PHC in France (aim A) and to develop a typology of this structuring in order to describe the level of geographic structuring and its potential for development (aim B). That will allow to better understand this process of territorial structuring and, in the future, to approach it using a tool which allows to describe territorial diversity.

## Methods

###  Study Design

 We conducted a sequential-exploratory mixed-method study^32^ with a qualitative comprehensive step using a multiple case approach (aim A) and a quantitative step as a multiple correspondence analysis (MCA) followed by a hierarchical clustering on principal components (HCPC) to elaborate the typology (aim B). The qualitative and quantitative steps provide complementary perspectives on the subject under study. Qualitative data allow for an in-depth exploration of underlying the territorial structuring of PHC, while quantitative data provide an overview.

###  Setting and Context

 It was first necessary to choose a relevant spatial unit or scale that could be qualitatively studied and where quantitative variables were available for the second step. We chose the “life-health territories” (*territoires de vie-santé*) as spatial unit. These territories, defined in a French regulatory text in 2021,^33^ divide mainland France into 2730 zones. The “life-health territory” is an aggregate of municipalities around a pole of infrastructures and services. This division aims to delimit the most tightly knit territory within which inhabitants have access to the healthcare infrastructures and services considered most common. Each municipality belongs to one life-health territory^[1]^. This division frees itself from administrative boundaries: municipalities belonging to different regions or counties (*départements*) may belong to the same life-health territory. The life-health territory is the geographic unit of reference used in incentive policies for the installation of GPs (above). The life-health territories are suitable for studying the territorial structuring of PHC given the HTPC generally organize across multiple municipalities within the same life-health territory.

###  Qualitative Comprehensive Step: PHC in France (Aim A)

####  Data Collection

 A multiple case study was conducted in 7 life-health territories with contrasting characteristics (Table 1) to understand the conditions for the emergence of territorial structuring of PHC. We chose a multiple case study approach because it allows for an in-depth exploration of the phenomenon of territorial structuring of PHC across different contexts.^34^ Each territory served as a unique case study, providing rich data on how PHC is organized and delivered. This approach enabled us to capture the complexity and diversity of PHC structures and practices across different settings, facilitating a more comprehensive understanding of the elements influencing territorial structuring. Qualitative data were collected by a single investigator (first author) over 4 months of fieldwork (January–April 2019). The material was collected through semi-structured interviews (n = 30) conducted with various PHC professionals (both leaders and non-leaders) (Supplementary file 2) and non-participatory observations (n = 7). Professionals were designated as “leader” if they had responsibilities in the governance of the HTPC. Indeed, the life-health territories were chosen because they included a HTPC or they were involved in the creation of a HTPC, in order to examine the structuring process as a whole with the interviewees. Participants were selected based on their degree of involvement in the process of the territorial structuring and their availability. It should be noted that at the time of conducting this qualitative fieldwork, the number of recognized HTPC was low, barely reaching several tens: only 15 HTPCs were recognized by the end of April 2019, 25 by the end of August 2019, and 39 by the end of 2019. Therefore, the 7 studied HTPC represented a significant proportion of the recognized HTPC at the end of April 2019 (>45%).

**Table 1:** Characteristics of the 7 Meso-Level Territories Explored

	**Population Covered**	**PHC Services and Territorial Context**	**Number of Independent HCPs**
Urban territories			
Territory 1	196 000 Inhabitants	HHs, healthcare centers, a LHC, 54 pharmacies	172 GPs, 24 midwives, 142 nurses, 158 physiotherapists
Territory 2	180 000 Inhabitants	A large multisite HH, 52 pharmacies	294 GPs, 18 midwives, 125 nurses, 214 physiotherapists
Peri-urban territories			
Territory 3	138 000 Inhabitants	Several HHs, 45 pharmacies	98 GPs, 11 midwives, 100 nurses, 92 physiotherapists
Territory 4	46 000 Inhabitants	An HH, a LHC, 13 pharmacies	35 GPs, 7 midwives, 41 nurses, 45 physiotherapists
Rural territories			
Territory 5	55 000 Inhabitants	An HH, a LHC, 21 pharmacies	49 GPs, 4 midwives, 75 nurses, 21 physiotherapists
Territory 6	28 500 Inhabitants	An HH and a multi-site one, a LHC, 8 pharmacies	21 GPs, 2 midwives, 36 nurses, 11 physiotherapists
Territory 7	20 500 Inhabitants	A recent HH, a LHC, 8 pharmacies	15 GPs, 2 midwives, 39 nurses, 24 physiotherapists

Abbreviations: PHC, primary healthcare; LHC, local health contract; GPs, general practitioners; HH, healthcare home; HCPs, healthcare professionals.

 An interview guide was used to conduct the interviews and explore the reasons that could explain the emergence of a structured territory as well as the barriers and enablers to the implementation of a HTPC in the territory (Supplementary file 2).

####  Data Analysis

 Interviews were recorded, transcribed, and then coded using the NVivo software, following informed consent. The material was analyzed, in French, using a “general inductive” approach^35^ which allows for the identification of categories that are then linked to the research objectives. Inductive analysis is particularly well-suited for analyzing data on exploratory research objects, for which the researcher does not have access to pre-existing categories in the literature. The key elements of the territorial structuring of PHC have thus been isolated and documented through verbatim responses each time. Each territory was also contextualized with a quantitative and qualitative descriptions of its healthcare supply (type and number of HCPs, type of multi-professional structures, date of their creation, types of practice of the HCPs), as reported in Table 1.

###  Quantitative Step: Develop a Territorial Typology of the Territorial Structuring of PHC in France (Aim B)

####  Data Collection

 Following the lessons from the qualitative phase, each life-health territory was characterized using variables, each describing a specific characteristic of the territory. These specific characteristics were grouped into three types of general features: the provision of PHC services within the territory, the evolution of PHC services over the past five years, and the longevity of PHC services. This differentiation of 3 general features directly followed the insights from the qualitative phase of the study and the determination of the key elements of PHC territorial structuring (See qualitative phase results).

 The corresponding data were obtained from 3 different sources: the permanent database of facilities (*base permanente des équipements*, BPE), the national file of health and social services (*fichier national des établissements sanitaires et sociaux*, FINESS), and the CLoterreS study database.

 The BPE database is a statistical database managed by INSEE, the French national institute of statistics and economic studies. It lists geographically a wide range of infrastructures and services, including healthcare services. The FINESS database is a national reference directory of multi-professional structures. It contains information on HHs, HCs, and HTPCs (location, date of creation). It is managed by the Ministry of Social Affairs and Health. The CLoterreS study is a study of LHCs, carried out by a Franco-Canadian research consortium.^29^ A database of all LHCs (with their location) signed in France was compiled from a comprehensive review: all the texts of the local contracts, accessible on the regional health agencies’ websites, were examined to identify all the municipalities where a LHC was deployed. The data used described the situation as of December 31, 2019. Data as of December 31, 2014 were used to describe the changes observed over the past 5 years.

 All variables were produced at the scale of the life-health territories. Quantitative variables available at the municipality scale were aggregated to be available at the life-health territory scale. They were then transformed into qualitative variables with categories based on the values of their median and/or quartiles. Thus, the variable describing the density of GPs was presented with 3 categories: Low (<63 GPs per 100 000 inhabitants), Medium ([63-102]) and High (>102). Similarly, the variable describing the density of HCPs (including GPs, midwives, physiotherapists, and nurses) was presented with 4 categories: Low (<34 HCPs per 100 000 inhabitants), Medium (]34-55]), High (]55-90]), Very high (>90). All other recodings are mentioned in the appendix material (Supplementary file 3). Binary variables describing the presence of a LHC, or a type of multi-professional structure (HH, HC, and HTPC) were obtained by considering the presence of each of these elements in at least one of the municipalities of a life-health territory considered. We ultimately described each territory using 16 categorical variables (Table 2).

**Table 2:** The 16 Variables Describing Each Life Health Territory, by “General Features” and “Specific Characteristics,” Data Sources, and Covered Years

**General Features**	**Specific Characteristics **	**Variables**	**Data sources**	**Year(s)**
PHC services provision	HCPs and services provision	Density of GPs (number of GPs per 100 000 inhabitants)	BPE database	2019
		Density of HCPs (number of GPs, midwives, physiotherapists, and nurses per 100 000 inhabitants)		
		Density of pharmacies (number of pharmacies per 100 000 inhabitants)		
	Multi-professional structures provision	Number of multi-professional structures (HHs, HCs)	FINESS database	
	Territorial context	Presence of a HTPC		
		Presence of LHC(s)	CLoterreS study	
PHC services evolution	HCPs’ and services evolution	Evolution of the density of GPs (number of GPs per 100 000 inhabitants)	BPE database	2014-2019
		Evolution of the density of nurses (number of nurses per 100 000 inhabitants)		
		Evolution of the number of midwives and physiotherapists (number of midwives and physiotharapists per 100 000 inhabitants)		
		Evolution of the density of pharmacies (number of pharmacies per 100 000 inhabitants)		
	Multi-professional structures provision evolution	Evolution of the number and nature of multi-professional structures provision	FINESS database	
	Territorial context evolution	Evolution of the presence of LHC(s)	CLoterreS study	
PHC services longevity	Multi-professional structures provision longevity	Longevity of HH provision	FINESS database	2009-2019
		Longevity of HC provision		
	Territorial context longevity	Longevity of HTPC provision		
		Longevity of LHC	CLoterreS study	

Abbreviations: PHC, primary healthcare; LHC, local health contract; GPs, general practitioners; HH, healthcare home; HC, healthcare center; BPE, base permanente des équipements; FINESS, fichier national des établissements sanitaires et sociaux; HCPs, healthcare professionals; HTPC, health territorial and professional community.

####  Data Analysis

 We performed an HCPC on the components derived from an MCA, at the territorial scale.^36^ Argüelles et al explain that the primary benefit of this approach, compared to using factorial analysis alone, is that it applies objective clustering techniques to the results of principal components analysis, resulting in a more robust cluster solution.^37^

 In a first step, the MCA was performed using the 16 categorical active variables qualifying the 2730 life-health territories. This step allowed us to describe the relationships between the variables and to summarize this information on synthetic factorial axes, also called principal components.^38^ Illustrative variables were integrated, but do not participate in the construction of the components.

 We kept the first seven factorial axes (components) by retaining the empirical criterion: axes prior to an inflection point in the eigenvalue decay curve, ie, to retain only the axes for which the second differences are positive (Catell’s elbow criterion).^39^ The detailed description of the correlations between the active variables and the first 7 factorial axes is presented elsewhere (Supplementary file 4). The Benzécri correction (presented in the manuscript) and the Greenacre correction were used to adjust the percentage of variance explained by each axis and can be found in the appendix materials (Supplementary file 4).

 In a second step, and based on the seven factorial axes described above, we performed the HCPC which aggregates the life-health territories two by two until coherent clusters were obtained. The clustering process was done using a mixed algorithm combining a Ward’s classification method with an aggregation around mobile centers (K-means).^37^ This method is used to obtain the greatest possible homogeneity within the clusters and the greatest possible heterogeneity between the clusters. For the choice of the number of clusters to be retained, a criterion of maximization of the proportion of inertia explained by the partition, ie, the ratio between the inter-class inertia and the total inertia, has been retained. Additional elements are presented in appendix materials (Supplementary file 4). Analysis was performed using R version 4.0 with factoextra, FactoMineR and sqldf packages.

## Results

###  Results of the Qualitative Comprehensive Step: The Key Elements of The Territorial Structuring

 The 7 explored territories had varied characteristics: 3 of them were rural territories, 2 were peri-urban territories, and 2 were urban territories (Table 1). The PHC provision, including the availability of multi-professional structures and the demographics of HCPs, varied from one territory to another: urban territories were generally better equipped than rural territories (Table 1). LHCs were many found in the explored territories.

 The analysis of the qualitative material highlighted that the long-standing presence of PHC provision in the territory constituted, in most cases, a prerequisite for territorial structuring, especially when this provision is organized (Table 3).

**Table 3:** The Key Elements of Territorial Structuring of Primary Healthcare (Mainland France, 2019)

**Key elements**	**Verbatim quotes**
Long-standing presence of a PHC provision in the territory	*“I find it difficult to see how one could create a HTPC in an area where there is no prior organization of primary care somewhere in this territory… there must be either a HH or one or more primary care teams.”*
Pre-territorial structuring through collaborations between HCPs or multi-professional structures	*“We can see the example of the HHs, for instance… that’s how it happened… it was a bit of pioneers who started the process, who got involved.”* *“Let’s start from the beginning, build it up, and then in the end we can put the roof on it, which is called a HTPC. Let’s not put the roof on it when we haven’t even built the foundations or walls!”*
Advanced level of territorial coordination within a HTPC	*“The HTPC is a mode of organization [of primary care], not a structure, and it’s not the same. One is patient-centered, and collective practice structures, such as HCs and HHs, are intended to meet the needs of their patient, while the HTPC […] targets a much larger population.”*
High number of independent and multidisciplinary HCPs in the territory	*“If you have a larger group, you more easily find two, three, four people motivated by a thing… whereas in an average HH… they are alone.”* *“We regularly have multi-professional meetings on topics that interest everyone. A collective of doctors and nurses... and then I go see the pharmacist, to work together.”*
Investment in territorial health through a LHC	*“There is a dynamic of LHC… I always said that we should get closer to the LHC [with the HTPC].”* *“I think it’s positive for a territory to have already had a LHC… it prepares the work for the HTPC on that.”* *“Well, everywhere there has been… because before the HTPC approach, there had been LHC.”*
Strong territorial demographic dynamic with the establishment of young professionals	*“Coordinating in private practice [is] showing young professionals that we know how to communicate with each other, that we are no longer isolated. […] When we do better than others, young professionals come to us!”* *“Replacement doctors often tell us that it’s great to arrive in a territory where there is already a network of professionals and where we already know who to ask if we need help. And that’s something that the young professionals have clearly show us.”* *“With 4 HHs created, we have over 17 GPs who have set up, and I don’t have the number for nurses, maybe around 15.”* *“They have had 7 new doctors who have settled in… in one or two years… whereas the area was completely deficient… and because they are organized themselves.”*

Abbreviations: PHC, primary healthcare; LHC, local health contract; GPs, general practitioners; HHs, healthcare homes; HCs, healthcare centers; HTPC, Health Territorial and Professional Communities; HCPs, healthcare professionals.

*“I find it difficult to see how one could create a HTPC in an area where there is no prior organization of primary care somewhere in this territory… there must be either a HH or one or more primary care teams” *(GP, Territory 1).

 This prerequisite appeared as a stage of ‘pre-territorial structuring’ at the micro level, characterized by collaborations around patient care among HCPs and/or multi-professional structures in the territory.

*“When we look at what happens with functioning or emerging HTPC, we can see that in 9 out of 10 cases, there is a strong and mature organized multi-professional healthcare home that has already solved its operational and patient management issues... One of the conditions for a HTPC to function is also to have a high level of primary care structuring” *(GP, Territory 6).

 The territorial structuring of PHC appeared to be most successful when a meso-level territorial coordination is organized among HCPs and/or multi-professional structures grouped in HTPC.

*“A HTPC is a gathering of healthcare professionals from a territory who wish to work together to improve the care of their patients and patient pathways” *(GP, Territory 2).

 One facilitating factor for territorial structuring was the number of independent HCPs in the territory: the greater their number, the more extensive and varied the cooperation, as groupings were facilitated. Conversely, implementing collaborations in a territory with a low number of HCPs would be more complex due to the need to gain the agreement of everyone involved.

 While the number of independent professionals matters, their diversity also appeared to be crucial in the process of territorial structuring, which aimed to be multi-professional by nature.

*“We regularly have multi-professional meetings on topics that interest everyone. A collective of doctors and nurses... and then I go see the pharmacist, to work together” *(Freelance nurse, Territory 1).

 Demographic dynamics should also be taken into account, as the process of structuring can be considered successful when young professionals decide to settle in the territory or when new multi-professional structures or teams form.

*“With 4 HHs created, we have over 17 GPs who have set up, and I don’t have the number for nurses, maybe around 15” *(GP, Territory 3).

 Lastly, it seemed that territorial structuring was facilitated when the territory had been invested in by public health, meaning when there was the presence of a LHC, particularly if it was longstanding.

*“We have many professionals in the HTPC who are motivated by the subject of health prevention and promotion, which we have started to do within the local health contract” *(Project manager, Territory 7).

 The qualitative variables selected to construct the typology were chosen based on these different key elements to reflect both the PHC provision present in the territory (in terms of HCPs and multi-professional structures), its evolution over the past 5 years, and its longevity (Table 2).

###  Results of the Quantitative Step

####  Components of the Multiple Correspondence Analysis

 The MCA reduced the information into components onto which the studied life-health territories could be projected (Supplementary file 4). The first seven components were subsequently used for the HCPC. The first component of territorial opposition (8.7% of the inertia; 30.0% after correction) based on the selected active variables was correlated with the presence of multi-professional structures, particularly HCs. The second differentiating component (5.9% of the inertia; 12.8% after correction) was more related to public health and the presence of an LHC. The third component of territorial opposition (4.9% of the inertia; 8.1% after correction) was again correlated with the availability of multi-professional structures but specifically with the recent presence of HHs in the area. The fourth component (4.5% of the inertia; 6.7% after correction) distinguished territories based on the presence or absence of a HTPC. The fifth component (4.1% of the inertia; 5.4% after correction) allowed differentiating territories based on the recent progression of the number of multi-professional structures. The sixth component (3.9% of the inertia; 4.6% after correction) opposed territories based on the longevity of the LHC. The final selected component (3.6% of the inertia; 3.9% after correction) synthesized oppositions between variables describing the demographics of HCPs in the area, including density of GPs.

####  A Four-Cluster Typology

 The typology resulting from the HCPC distinguishes four categories of life-health territories: (*i*) under-structured (weakly structured) or unstructured territories, (*ii*) territories with potential for structuring, (*iii*) territories in the way for structuring, and yet (*iv*) structured territories.

 The first category comprises 1054 life-health territories, representing 38.6% of the mainland territories (Table 4). These predominantly peri-urban territories have a total population of nearly 17 million. These territories are characterized by the absence of multi-professional structures and a relatively stable or declining professional demographic over the past five years. They also have the lowest number of LHCs (only 26.1% of territories in this category compared to 36.2% for all mainland territories). Therefore, these life-health territories can be described as non-structured or weakly structured territories.

**Table 4:** Characteristics of the Clusters of the Territorial Structuring of Primary Healthcare (Mainland France, 2019)

	**Total**	**Cluster 1**	**Cluster 2**	**Cluster 3**	**Cluster 4**	***P*** ** Value**^a^
	**N = 2730 (100)**	**n = 1054 (38.6)**	**n = 946 (34.7)**	**n = 691 (25.3)**	**n = 39 (1.4)**	
Population (in millions of inhabitants)	65.9	16.9	24.4	23.1	1.5	NA
Territorial characteristics, No. (%)						<.001
Urban	515 (18.9)	188 (17.8)	201 (21.2)	119 (17.2)	7 (17.9)	
Peri-urban	536 (19.6)	288 (27.3)	172 (18.2)	71 (10.3)	5 (12.8)	
Rural	1679 (61.5)	578 (54.8)	573 (60.6)	501 (72.5)	27 (69.2)	
Density of GPs (number of GPs per 100 000 inhabitants), mean (SD)	87.2 (42.0)	86.7 (47.1)	89.1 (41.6)	85.4 (34.3)	84.3 (27.0)	0.5
Density of non-GPs HCPs (number per 100 000 inhabitants), mean (SD)	270.5 (155.7)	287.7 (171.4)	260.5 (124.7)	260.4 (169.0)	227.4 (89.7)	0.001
Proportion of GPs among the HCPs (%), mean (SD)	26.0 (10.0)	25.1 (10.0)	26.7 (10.0)	26.5 (10.0)	28.0 (10.0)	<.001
Number of pharmacies per 100 000 inhabitants, mean (SD)	35.5 (14.2)	33.1 (14.0)	35.9 (14.9)	38.4 (13.1)	37.3 (13.1)	<.001
Number of multi-professional structures per 100 000 inhabitants, mean (SD)	6.8 (24.6)	0.0 (0.0)	10.2 (21.1)	12.1 (40.8)	12.5 (7.7)	<.001
Number of multi-professional structures, No. (%)						<.001
None	1062 (38.9)	1054 (100)	0 (0.0)	2 (0.3)	6 (15.4)	
1 or 2	1244 (45.6)	0 (0.0)	767 (81.1)	460 (66.6)	17 (43.6)	
More than 2	424 (15.5)	0 (0.0)	179 (18.9)	229 (33.1)	16 (41.0)	
Proportion of HHs among the multi-professional structures (%), mean (SD)	50.0 (42.9)	-	51.5 (44.6)	56.9 (40.8)	32.0 (23.4)	<.001
Presence of a HTPC, No. (%)	39 (1.4)	0 (0.0)	0 (0.0)	0 (0.0)	39 (100)	<.001
Presence of an LHC, No. (%)	987 (36.2)	275 (26.1)	0 (0.0)	691 (100)	21 (53.8)	<.001
Evolution of the density of GPs (%), mean (SD)	-1.6 (26.1)	-1.4 (25.4)	-0.6 (26.5)	-3.0 (26.8)	-4.1 (19.5)	<.001
Evolution of the density of non-GPs HCPs (%), mean (SD)	20.3 (28.3)	19.2 (25.5)	20.9 (22.7)	20.8 (37.6)	24.2 (28.3)	0.6
Evolution of the multi-professional structures provision, No. (%)						NA
No multi-professional structure	1062 (38.9)	1054 (100)	0 (0.0)	2 (0.3)	6 (15.4)	
Stability (no variation)	613 (22.5)	0 (0.0)	361 (38.2)	243 (35.2)	9 (23.1)	
New provision based on HH	535 (19.6)	0 (0.0)	317 (33.5)	207 (30.0)	11 (28.2)	
Development of HHs	127 (4.7)	0 (0.0)	52 (5.5)	70 (10.1)	5 (12.8)	
Development of HCs	253 (9.3)	0 (0.0)	154 (16.3)	97 (14.0)	2 (5.1)	
Development of HHs and HCs	140 (5.1)	0 (0.0)	62 (6.6)	72 (10.4)	6 (15.4)	
Longevity of LHC, No. (%)						<.001
None	1743 (63.8)	779 (73.9)	946 (100)	0 (0.0)	18 (46.1)	
Opened less than 5 years ago	356 (13.0)	116 (11.0)	0 (0.0)	234 (33.9)	6 (15.4)	
Opened 5 years ago or more	631 (23.1)	159 (15.1)	0 (0.0)	457 (66.1)	15 (38.5)	

Abbreviations: GPs, general practitioners; LHC, local health contract; HH, healthcare home; HCs, healthcare centers; SD, standard deviation; NA, not applicable; HCPs, healthcare professionals; HTPC, health territorial and professional community. ^a^Fisher’s exact test for qualitative variables and Kruskal-Wallis test for quantitative variables.

 The second category obtained consists of 946 life-health territories, accounting for 34.7% of the studied mainland territories. These territories encompass 24.4 million inhabitants and are predominantly rural (60.6%). They are characterized by a significant positive evolution in the density of HCPs and a lesser decrease in GP density. The vast majority of these territories (81.1%) have 1 to 2 multi-professional structures. However, none of these territories have LHC. This category appears to be marked by isolated professional dynamics (arrival of new professionals in the last 5 years, creation of HHs or HCs in the territory) despite a low investment in local public health. The territories involved are thus classified as non-structured but with a high potential for structuring.

 The third category of the typology comprises 691 life-health territories (25.3%). These territories are predominantly rural (72.5%) and encompass just over 23 million inhabitants. All territories in this category have a LHC, and almost all of them (99.7%) have at least one multi-professional structure. One-third of them have more than 2 multi-professional structures, mainly represented by HCs, with nearly half of the territories having newly established HCs. These territories demonstrate a strong commitment to local public health, and there is a dynamic creation of new multi-professional structures, perhaps aiming to slow down the decline in medical demography, which is nearly twice the mainland France average. This category encompasses territories considered to be in the process of structuring.

 Finally, the fourth category brings together a few life-health territories (n = 39, 1.4%) where a HTPC is identified. These territories encompass 1.5 million people and are predominantly rural. The number of multi-professional structures in these territories is generally high, despite a decline in medical demography. Conversely, the evolution of non-physician HCPs’ demography in these territories over the past 5 years is positive. These territories, with an HTPC, are classified as structured territories.

 The mainland map illustrating the level of territorial structuring by life-health territories in 2019 shows significant disparities (Figure). Territories with an HTPC are highly prevalent in the central region of France (Centre-Val de Loire region) and around several mainland areas. Territories with potential for structuring are notably present in rural geographical areas, such as the borders of Île-de-France region.

**Figure:**
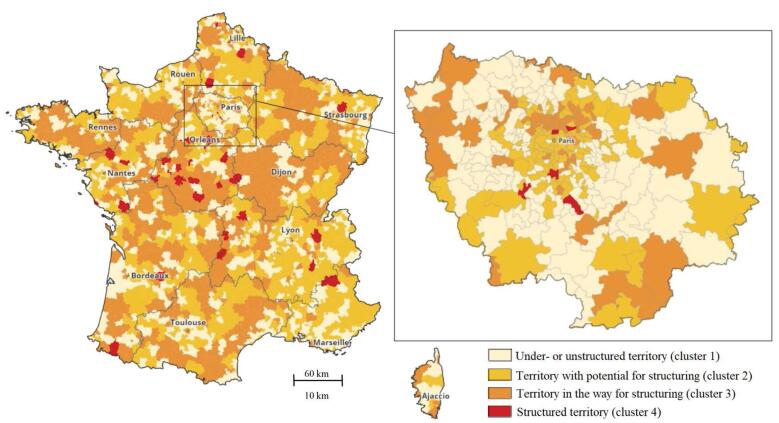
Cartography of the 4 Categories of Life-Health Territories. Focus on the Ile-de-France, Paris region. Mainland France, 2019.

## Discussion

 In this work, we have determined the key elements of the territorial structuring of the PHC (aim A) and developed a first graduated typology describing the degree of territorial structuring of PHC in France (aim B). We have been able to highlight that PHC is differently structured by distinguishing life-health territories with limited or no structuring, with potential for structuring, in the way for structuring, and that are already structured. This difference reflects varied territorial practices and dynamics, which may be longstanding.

 This original work falls within the field of health services research and geographical analysis based on spatial typologies,^40–42^ particularly in the study of healthcare systems.^43,44^ It focuses on the phenomenon of territorial structuring of PHC, which, in the French context of a widespread shortage of ambulatory medical resources and a specific decline in the GPs’ demographic,^45^ appears as a possible strategy to ensure the continuity and effectiveness of healthcare for the population.^26,46^ The independent nature of medical practice justifies delving into this process of territorial structuring, as it cannot be decreed but requires the professionals’ adherence and progressive implementation. Thus, through a more in-depth understanding of the mechanisms involved in the territorial structuring of PHC and a more precise knowledge of the territorial structuring, this study opens up avenues for reflection on the ability of the French PHC system to address the challenge of the decline in medical demographics.^25,26^ This challenge is shared by many countries facing limitations in terms of healthcare human resources.^47^ Moreover, the recent COVID-19 crisis has demonstrated the value of this territorial structuring and coordinated organizational models in enabling PHC actors to provide rapid and adapted responses.^48,49^ The discussion surrounding the reinforcement of the territorial structuring of PHC also focuses on the resilience capacity of healthcare organizations, as suggested by Orvik et al through the concept of “organizational health.”^50^

 The qualitative phase of our mixed-methods study allowed us to emphasize that the process of territorial structuring of PHC is a long-term process. While it is visible today with the emergence of HTPCs in certain territories, it remains a product of history and does not occur without investment from HCPs in the territory. This observation has been described in the literature, particularly in case studies where authors explain the significance of past collaborations.^51^ It is not uncommon to see an HH emerge in a territory that foreshadows a HTPC.^52^ The transformation of local healthcare provision can only be understood in relation to its professional and territorial environment (provisions available) and over time (evolution and longevity). While these observations help us better understand the phenomenon of territorial structuring of PHC, they also highlight its strong connection to the territorial context. Therefore, it is important to note that the typology we have developed cannot fully synthesize or account for all this variability at the territorial level.

 The results of our typology highlight disparities between regions and, more importantly, at the sub-regional level. This regional disparity could reflect varying levels of support for territorial structuring of PHC by regional health agencies, which, although strong national directives may have been issued from the Ministry of Health, remain decision-makers in the modalities of HTPCs deployment.^53^ For instance, the high presence of HTPCs in the Centre-Val de Loire region can be partly explained by the explicit delegation given by the regional health agency to the regional representatives of HCPs (*union régionale des professionnels de santé*) to deploy HTPCs in all territories (above).

 The territorial structuring of PHC does not seem to follow a known cartographic division, except for the fact that major urban areas mainly gather territories “with potential for structuring” (Figure). Conversely, a lower level of territorial structuring (“under- or unstructured territories”) is observed in the immediate outskirts of large cities and in peri-urban areas closest to the metropolises. This concentration effect in urban areas is described in other contexts as well.^54^ This contrast is particularly visible in the Île-de-France region, where the level of structuring varies as one moves away from the center of Paris, while at the region’s boundaries, where medical demography is lower, the territories show higher levels of structuring. This can be partly explained by the fact that some municipalities choose to subsidize the construction of HHs or HCs to revitalize and make their territories more attractive.^55^ In this case, the structuring is either announced (“potential for structuring”) or in progress (“in the way for structuring”). Territories considered already structured, ie, with a HTPC, are scattered and unevenly distributed nationwide.

 The typology we have developed aligns with other typologies proposed in previous studies, which have primarily focused on qualitative descriptions of territories regarding aspects such as multi-professional collaboration, intersectoral coordination, and local integration.^56,57^ We have identified 3 typologies that we want to compare with our typology. The first is the one developed in 2003 by Beaulieu et al in the context of the restructuring of Canadian health services.^58^ Beaulieu thus proposes a classification of PHC services into 4 models: two “professional models,” one called “contact” and the other called “coordination,” and two “community models,” one “integrated” and the other “non-integrated.” The professional models aim to provide medical services to patients who present themselves or according to a consultation logic (contact model) where GPs are rarely associated with other HCPs, or according to a service logic (coordination model), with work often done in teams and remuneration on a capitation basis. The community models, on the other hand, aim to improve the health status of populations in a given territory. The non-integrated model incorporates no integration mechanism, services being offered without collaboration with other components of the healthcare system, unlike the integrated model which aims to promote integration. This four-category typology, with a form of progression between independent practice, collaboration, and care integration, is similar the one we propose, in which the highest level of structuring is realized through an integration logic driven by HTPC. However, the territorial dimension, which is at the core of our typology, is absent from the models proposed by Beaulieu.

 The second typology we want to discuss is the one developed by Rodriguez Duarte, who conducted a mixture model analysis among 368 interprofessional PHC teams in Quebec to develop an organizational typology of interprofessional teams.^59^ She distinguishes five profiles as follows: “Very small team, private, regular, high level of partnership, and balanced team” (n = 99; 26.9%); “Small, private, regular, modest partnership, and balanced team” (n = 101; 27.5%); “Medium, public, academically oriented, low partnership, practitioner-oriented team” (n = 58; 15.8%); “Large, private, regular, very low partnership, and balanced team” (n = 50; 13.6%); and “Very large, private, mixed, very low partnership, and balanced team” (n = 60; 16.3%). Similar to the previous one, this typology differs from ours in that it does not incorporate the territorial dimension. However, it emphasizes that collaboration logics are relatively distinct from the size of the teams considered, as we observe in our typology where certain territories deemed “potential for structuring” have relatively high densities of professionals without high levels of structuring.

 Finally, a classification proposed by Fournier et al in 2021 presents points of similarity with the typology we have developed.^60^ We can thus link the territories they describe as having “active collaboration and minimal coordination” to those we have identified as territories with “potential for structuring,” considering the emerging collaborations and observed professional dynamics. The territories in which they observe “well-developed coordination, but limited collaboration” exhibit characteristics similar to those we described in our category “in the way for structuring.” Finally, the territories with “local collaboration, coordination, and territorial integration” can be considered as already structured territories. In fact, Fournier et al have accurately described the role of HTPC in these integrated territories. Our quantitative and systematic approach thus complements qualitative approaches that generally focus on a limited number of territories. Indeed, unlike these previous typologies, our approach offers a comprehensive examination of territorial structuring across all territories of mainland France, providing a more expansive and nuanced understanding of PHC dynamics.

 However, our work has certain limitations. Firstly, it is important to highlight that the quality of the data used suffers from an information bias, which, although reduced (less than 5% of life-health territories), is still present. The geolocation database of HHs and HCs used is not exhaustive because it is based on an administrative census that occurs after the creation of the multi-professional structure. Administrative registration may occur several months after the establishment of the structure, and some structures may never be included in the census, especially if they do not require specific funding. Therefore, some dynamics may have been overlooked in certain territories due to this information bias. Moreover, in our analysis, we did not use an accessibility indicator, such as the APL established at the municipal level,^61^ considering that our approach, more focused on professionals, did not require assessing the adequacy of supply to demand for care but rather quantifying it and studying its evolution and history. Additionally, it should be noted that the indicators used are annualized indicators, and the typology was not performed synchronously. This calls for an update of the typology and periodic repetition of this work, especially at a distance from the COVID-19 epidemic, which has had a significant impact on PHC.^62^ Furthermore, HTPCs continue to be deployed across the territory. In June 2023, there were 444 HTPCs recognized by health insurance. Another limitation concerns the chosen territorial delineation, which, although relevant considering the indicators used, only allows for mapping of mainland France. Furthermore, this territorial framework is specific to France and limits generalizations. However, the methodology used, involving a qualitative exploratory phase followed by an analytical phase of geo-epidemiology, could be replicated in a different context. We did not assess spatial autocorrelation within our typology. Future research could explore this aspect to better understand spatial patterns of the territorial structuring.

 Despite these limitations, our typology has several advantages. First and foremost, it provides a comprehensive categorization of the territorial structuring of PHC throughout France, which has been recently developed with the deployment of HTPCs, and therefore needs to be understood in its entirety.^26^ This typology helps better understand the diversity of organizational configurations of PHC services across different territories in France providing a description that enhances our understanding of this territorial structuring process. By identifying and characterizing this territorial structuring, policymakers and HCPs can better target the specific needs of each territory and tailor health policies accordingly. Additionally, a clear typology can serve as a framework for comparing the performance of different territorial structuration lead by organizational models. It can also facilitate public health research by providing a basis for comparative studies and trend analyses in PHC systems. Indeed, this typology could be reused as a potential explanatory factor in epidemiological studies where an understanding of territorial characteristics is important.^63,64^ The consideration of territorial aspects is also crucial in impact assessments at the territorial level.^65^ In intervention studies, our typology could ensure comparability between territories that receive the intervention and those that do not. This could be particularly relevant for the implementation of public health policies at the territorial level and the study of their intrinsic effects, aiming to minimize territorial variability as much as possible. For instance, while it is anticipated that HTPC may play a facilitating role in attracting young HCPs to the territory, it would be worthwhile to conduct a study examining the effects of territorial structuring of primary care, particularly through HTPC, on professional recruitment, as has been done with HHs.^66^ Finally, the typology serves as a valuable tool for public health authorities, enabling them to monitor the progression of territorial structuring and enhance efforts tailored to specific areas.

 Lastly, our typology was established by considering indicators related to PHC as well as indicators related to territorial public health (presence, longevity, and dynamics of LHCs in the territory). At the territorial level, the functions of public health and PHC intersect and hybridize, within an approach centered on the population of the territory.^67,68^ The significant presence of LHCs in territories considered to be more strongly structured. This result suggests that the development of local public health, through such contracts, can facilitate the territorial structuring of PHC through the actions undertaken like: recognizing the role of primary care in local healthcare provision, creating multiprofessional structures to maintain an adequate primary care provision in the territory, and raising awareness among professionals about population health issues. Although causal relationships cannot be demonstrated with our work, the presence of LHCs alongside a dynamic in PHC structuring tends to underscore the value of an alliance between primary care and public health at the meso-level. Public health actors must be able to support this process in collaboration with PHC actors, whose healthcare-focused culture could be challenged in addressing broader population-level issues.^68,69^

## Conclusion

 Interest in the territorial structuring of PHC aligns with a dual interest: firstly, the challenges of meso-level organization within the healthcare system and the need to develop more integrated care; and secondly, the territorial organizational configurations that must enable it. While the determinants, as well as the effects, of care coordination once established are well-documented, the key elements leading to territorial coordination remain to be specified. Providing a typology of territorial structuring of PHC should thus better grasp this phenomenon and ultimately study its effects in terms of healthcare access, quality of care, and its impact on medical resources. While territorial structuring is now widely implemented through the deployment of HTPCs in France, it takes on varied forms across countries. This undoubtedly calls for an in-depth comparative analysis of meso-level territorial organizations in PHC.

## Acknowledgements

 Yann Le Bodo and Eric Breton, from the EHESP – French school of Public health, for their assistance in developing this typology through the sharing of data on LHCs from the CLoterreS study.

## Ethical issues

 The study protocol and data collection were declared to the French Commission on Information Technology and Liberties, CNIL (N° 2229624).

## Competing interests

 Authors declare that they have no competing interests.

## Endnotes

 [1] For the three largest French cities (Paris, Lyon, Marseille), the life-health territories correspond to the districts (arrondissements): 20 for Paris, 16 for Marseille, and 9 for Lyon.

## Supplementary files


Supplementary file 1. Vocabulary.

Supplementary file 2. Qualitative Comprehensive Step.

Supplementary file 3. Recoding Variables.

Supplementary file 4. Multiple Correspondence Analysis and Hierarchical Clustering on Principal Components.
