# Supplementary material for: How Primary Healthcare Sector is Organized at the Territorial Level in France? A Typology of Territorial Structuring
Source: Int J Health Policy Manag. 2024 Jun 11;13:8231. doi: 10.34172/ijhpm.2024.8231 (PMC11270609; doi:10.34172/ijhpm.2024.8231)
Supplement: Supplementary file 1 — Vocabulary. [file ijhpm-13-8231-s001.pdf]

Article title: How Primary Healthcare Sector is Organized at the Territorial Level in France? A Typology of Territorial Structuring

Journal name: International Journal of Health Policy and Management (IJHPM)

Authors' information: Sylvain Gautier[1,2]*, Loïc Josseran[1,2]

[1]Research Center in Epidemiology and Population Health, Primary Care and Prevention Team, Inserm U1018, Université Paris-Saclay, UVSQ, Villejuif, France.

[2]Department of Hospital Epidemiology and Public Health, Raymond Poincaré Hospital, GHU Université Paris-Saclay, AP-HP, Garches, France.

***Correspondence to:** Sylvain Gautier; Email: sylvain.gautier@uvsq.fr

Citation: Gautier S, Josseran L. How primary healthcare sector is organized at the territorial level in France? A typology of territorial structuring. Int J Health Policy Manag. 2024;13:8231. doi:10.34172/ijhpm.2024.8231

Supplementary file 1. Vocabulary

Table S1.1: Vocabulary.

French word	English word translation	Description
CPTS – communautés professionnelles territoriales de santé	HTPC - health territorial and professional communities	Network of healthcare professionals who work together to improve the coordination and quality of care for patients in a specific geographic area. HTPCs are created and led by healthcare professionals themselves, and they are funded by the national health insurance.
MSP – maisons de santé pluriprofessionnelles	HH – healthcare home	French healthcare facility that brings together different healthcare professionals under one roof. HHs are designed to improve the coordination and quality of care for patients by making it easier for them to see different healthcare professionals in one place.
CDS – centres de santé	HC – healthcare center	French healthcare facility that provides primary care to patients. It is similar to a community health center in the United States.
CLS – contrat local de santé	LHC – local health contract	French agreement between the regional health agency and a local authority (municipality in general) to improve the health of the population in a specific geographic area. LHCs are typically developed and implemented over a period of five years.
ARS – agence régionale de santé	Regional health agency	French public health agency that is responsible for the regional implementation of health policy in France. There are 18 ARS in France, each covering a different region.
GHT – groupement hospitalier de territoire	Territorial hospital group	French regional hospital group that is a voluntary grouping of public hospitals in a specific geographic area. GHTs were created in 2016 as part of a reform of the French healthcare system.
ROSP – rémunération sur objectif de santé publique	Remuneration based on public health objectives	It is a system of financial incentives that is used to encourage healthcare professionals to improve the quality of care for patients.
CAPI - contrat d'amélioration des pratiques individuelles	Contract for improvement of individual practice	It is a voluntary agreement between a French general practitioner and the National Health Insurance Fund to improve the quality of care for patients.
URPS – union régionale des professionnels de santé	Regional union of healthcare professionals	It represent liberal healthcare professionals according to their sector of activity. They were created by the Law reforming hospitals and concerning patients, health, and territories of July 21, 2009.
CPAM – caisse primaire d'assurance maladie	Primary health insurance fund	Ensures close relations with the beneficiaries of the National Health Insurance Fund.
