# Supplementary material for: How Primary Healthcare Sector is Organized at the Territorial Level in France? A Typology of Territorial Structuring
Source: Int J Health Policy Manag. 2024 Jun 11;13:8231. doi: 10.34172/ijhpm.2024.8231 (PMC11270609; doi:10.34172/ijhpm.2024.8231)
Supplement: Supplementary file 2 — Qualitative Comprehensive Step. [file ijhpm-13-8231-s002.pdf]

Article title: How Primary Healthcare Sector is Organized at the Territorial Level in France? A Typology of Territorial Structuring

Journal name: International Journal of Health Policy and Management (IJHPM)

Authors' information: Sylvain Gautier[1,2]*, Loïc Josseran[1,2]

[1]Research Center in Epidemiology and Population Health, Primary Care and Prevention Team, Inserm U1018, Université Paris-Saclay, UVSQ, Villejuif, France.

[2]Department of Hospital Epidemiology and Public Health, Raymond Poincaré Hospital, GHU Université Paris-Saclay, AP-HP, Garches, France.

***Correspondence to:** Sylvain Gautier; Email: sylvain.gautier@uvsq.fr

Citation: Gautier S, Josseran L. How primary healthcare sector is organized at the territorial level in France? A typology of territorial structuring. Int J Health Policy Manag. 2024;13:8231. doi:10.34172/ijhpm.2024.8231

Supplementary file 2. Qualitative Comprehensive Step

Table S2.1: Semi-structured interview guide.

Table S2.2: List of individuals interviewed in the 7 territories studied.

Table S2.3: Translation of the verbatims.

Table S2.4: *NVivo* nodes – "coding tree".

Table S2.1: Semi-structured interview guide

Intro.	1. What does an HTPC mean to you?<br>2. In your opinion, what should an HTPC respond to? Do you think this is useful?
Axis 1: Engagement Drivers	3. In your opinion, what justifies the need for an HTPC in your territory? What will this be used for in your territory?<br>4. In what way are you personally involved in the emergence / construction / development of the HTPC? Can you remind me of the history of this project?<br>5. Why did you decide to get involved in the emergence / construction / development of the HTPC?<br>6. What health problem(s) (or others – quality, access, security, social), which would be relevant to question at the territorial level of your HTPC, do you identify? How do you think you can solve it?<br>7. Who are the actors (professionals, establishments, associations, local authorities, etc.) involved in your HTPC? How did they decide to participate in the life / construction / development of the HTPC? How do you manage to work together and what difficulty(s) do you encounter?<br>8. Who are your different partners (outside the HTPC)?<br>9. In your opinion, what brings you together within this HTPC? What could divide you?<br>10. What benefit(s) do you personally derive from your involvement in the HTPC? What is your interest in participating?<br>11. In your opinion, what is(are) the strength(s) and weakness(es) of your HTPC?
Axis 3: Action resources	12. How is the HTPC organized? Do you have meetings on this scale to discuss structural issues or difficult situations identified?<br>13. If applicable, who leads these meetings (a leader, an administrator, a third-party methodologist, etc.)?<br>*By "action" we mean any form of intervention designed at the HTPC level (information sharing, training, protocols, financing, creation of a service or activity for users, etc.)*<br>14. If you have done so, what action(s) have you implemented at the HTPC level? What problem(s) does it aim to address?<br>15. Which HTPC professional(s) were involved in these actions?<br>16. If applicable, which other professional(s) did you have to call upon to carry out these actions? For what reasons)?<br>17. In your opinion, what skill(s) are you lacking within the HTPC? (project management, animation, administrative management, gathering information, etc.)<br>18. Which partner(s) do you work with (ARS*, local authorities and elected officials, professional representations, etc.)? With each of these partners, how are exchanges carried out? For example, do you have a stable contact?
Axis 4: Human resources	19. Has the HTPC been or is the opportunity for sharing of human resources (secretarial, coordination, administrative, etc.)? How is it possible?<br>20. What place do specialist doctors (hospital and community) occupy within the HTPC? Are they all present within the HTPC? What are the relationships like with specialists outside the HTPC? Are they different from those that exist within the HTPC?<br>21. Who, in your opinion, is best able to manage, manage, organize and direct the professional collective of the HTPC?
Axis 5: Governance and financing	22. What place do establishments and services occupy within the HTPC? Are they integrated or apart? Do they participate in defining objectives?<br>23. What is (are) your relationship with the ARS*? during? Health Insurance / CPAM*? the URPS*?<br>24. How are decisions made within the HTPC? Who takes them?<br>25. Who is in charge of the HTPC budget? Who carries out the financial arbitrations if necessary?
Others	26. What does "having a sense of collective" mean to you?<br>27. In your opinion, what is "population responsibility"?<br>28. How do you envisage the HTPC deployment process?<br>29. What do you think is the role of the ARS* in the system? of the URPS*?

* The meanings of the abbreviations in English are provided in Appendix S2.1.

Table S2.2: List of individuals interviewed in the 7 territories studied.

No.	Occupation	Sex	Age range	Territory
1	General practitioner	H	60-70 years old	Territory 1
2	General practitioner	F	30-40 years old	Territory 2
3	Project manager	F	20-30 years old	Territory 3
4	Public health doctor	F	50-60 years old	
5	Mission manager	H	40-50 years old	Territory 1
	Project manager	F	30-40 years old	
6	Project Manager	H	40-50 years old	Territory 1
7	General practitioner	H	50-60 years old	Territory 2
8	CPTS Coordinator	F	50-60 years old	
9	General practitioner	H	30-40 years old	Territory 1
10	General practitioner	F	50-60 years old	
	Freelance nurse	F	30-40 years old	
	Freelance nurse	F	20-30 years old	
11	General practitioner	F	50-60 years old	Territory 3
12	Public health doctor	F	50-60 years old	Territory 3
13 & 14	CPTS Coordinator	F	30-40 years old	Territory 4
	General practitioner	H	40-50 years old	
15	Deputy Director	F	40-50 years old	Territory 5
	Project manager	F	50-60 years old	
16	General practitioner	F	50-60 years old	Territory 6
17	General practitioner	H	50-60 years old	Territory 3
18	Geographer	F	30-40 years old	Territory 6
19	General practitioner	H	50-60 years old	
20	CPTS Coordinator	F	30-40 years old	
21	Neurologist	H	50-60 years old	
22	Deputy Director	F	30-40 years old	Territory 4
23	Freelance nurse	F	50-60 years old	Territory 5
24	General practitioner	H	60-70 years old	
25 & 26	General practitioner	H	30-40 years old	
	Medical extern	H	20-30 years old	
27	Project manager	F	30-40 years old	Territory 7
28	Pharmacist	F	30-40 years old	
29	General practitioner	F	50-60 years old	
30	MSP Coordinator	F	30-40 years old	

Table S2.3: Translation of the verbatims.

French verbatim	English translation
« Je vois difficilement comment on pourrait créer une CPTS sur un territoire où il n'y a pas à certains endroits de ce territoire une organisation a minima des soins primaires … il faut soit une MSP ou une ou plusieurs équipes de soins primaires. » (01)	*"I find it difficult to see how one could create a HTPC in an area where there is no prior organization of primary care somewhere in this territory… there must be either a HH or one or more primary care teams."*
« On voit les maisons de santé par exemple... c'est... les maisons de santé c'est comme ça que ça s'est fait... c'est un peu des pionniers qu'ont lancé un peu la démarche, qui se sont investis. » (16)	*"We can see the example of the HHs, for instance… that's how it happened… it was a bit of pioneers who started the process, who got involved."*
« Partons du début, construisons et puis à la fin on mettra le toit qui s'appelle CPTS. Ne mettons pas le toit alors qu'on n'a même pas monté ni les fondations ni les murs de la maison. » (02)	*"Let's start from the beginning, build it up, and then in the end we can put the roof on it, which is called a HTPC. Let's not put the roof on it when we haven't even built the foundations or walls!"*
« La CPTS est un mode d'organisation [des soins primaires] ce n'est pas une structure et le cadre n'est pas le même. L'un c'est la patientèle, les structures d'exercice collectif, donc centres de santé et maisons de santé pluriprofessionnelles, ont vocation à répondre aux besoins de leur patientèle, alors que la CPTS […] vise une population beaucoup plus large » (5)	*"The HTPC is a mode of organization [of primary care], not a structure, and it's not the same. One is patient-centered, and collective practice structures, such as HCs and HHs, are intended to meet the needs of their patient, while the HTPC […] targets a much larger population."*
« Si vous avez un groupe plus large, vous trouvez plus facilement deux, trois personnes, quatre personnes motivées par un thème... alors que dans une MSP moyenne… ils sont seuls » (22)	*"If you have a larger group, you more easily find two, three, four people motivated by a thing… whereas in an average HH… they are alone."*
« On a donc très régulièrement des réunions pluriprofessionnelles... donc sur une thématique qui intéresse tout le monde. Un collectif de médecins et d'infirmières… et après je vais voir le pharmacien et on travaille ensemble » (10)	*"We regularly have multi-professional meetings on topics that interest everyone. A collective of doctors and nurses... and then I go see the pharmacist, to work together."*
« il y a une dynamique CLS... moi j'ai toujours dit qu'on devait se rapprocher des CLS » (21)	*"There is a dynamic of LHC… I always said that we should get closer to the LHC [with the HTPC]"*
« je pense que c'est positif pour un territoire d'avoir déjà eu un CLS... ça prépare le travail pour la CPTS sur ça. » (16)	*"I think it's positive for a territory to have already had a LHC… it prepares the work for the HTPC on that."*
« Bon partout où il y a eu... parce que en amont de la démarche CPTS, il y avait eu des CLS » (20)	*"Well, everywhere there has been… because before the HTPC approach, there had been LHC."*
« Une volonté de montrer qu'on sait se coordonner en libéral pour montrer aux jeunes qu'on sait se parler entre nous, qu'on n'est plus isolés. […] quand on fait mieux que les autres, les jeunes y viennent ! » (04)	*"Coordinating in private practice [is] showing young professionals that we know how to communicate with each other, that we are no longer isolated. […] When we do better than others, young professionals come to us!"*
« Les remplaçants ils nous disent souvent "c'est bien d'arriver dans un territoire où il y a un réseau de professionnels qui existe déjà et où on sait déjà vers qui adresser en cas de besoin... d'aide". Et ça on l'a bien... enfin... ils nous l'ont bien montré les jeunes médecins » (03)	*"Replacement doctors often tell us that it's great to arrive in a territory where there is already a network of professionals and where we already know who to ask if we need help. And that's something that the young professionals have clearly show us."*
« Avec quatre maisons de santé qui sont créées. On a plus de dix-sept médecins généralistes qui se sont installés, les infirmiers je n'ai pas le nombre... peut-être une quinzaine » (12)	*"With 4 HHs created, we have over 17 GPs who have set up, and I don't have the number for nurses, maybe around 15"*

French verbatim	English translation
« Ils ont 7 nouveaux médecins qui se sont installés... en un ou deux ans... alors que le territoire était complètement déficitaire... et que... parce qu'ils sont organisés » (19)	*"They have had 7 new doctors who have settled in… in one or two years… whereas the area was completely deficient… and because they are organized themselves"*
« Quand on regarde ce qui se passe au niveau des CPTS qui fonctionnent celles qui fonctionnent ou celles qui émergent, on voit bien qu'il y a 9 fois sur 10 il y a une MSP forte mature organisée qui a déjà enfin... qui a déjà résolu ses problèmes de fonctionnement, ses problèmes de gestion de patientèle… une des conditions aussi pour qu'une CPTS fonctionne c'est aussi d'avoir un niveau de structuration des soins primaires important » (20)	*"When we look at what happens with functioning or emerging HTPC, we can see that in 9 out of 10 cases, there is a strong and mature organized multi-professional healthcare home that has already solved its operational and patient management issues... One of the conditions for a HTPC to function is also to have a high level of primary care structuring."*
« Une CPTS c'est un regroupement de professionnels de santé d'un territoire qui souhaitent travailler ensemble pour améliorer la prise en charge de leurs patients et les parcours patients » (07)	*"A HTPC is a gathering of healthcare professionals from a territory who wish to work together to improve the care of their patients and patient pathways."*
« on a quand même beaucoup de professionnels dans les CPTS qui sont motivés par ce sujet prévention-promotion de la santé. Ce que du coup nous on a commencé à faire finalement au sein du contrat local de santé » (27)	*"We have many professionals in the HTPC who are motivated by the subject of health prevention and promotion, which we have started to do within the local health contract."*

Table S2.4: *NVivo* nodes – "coding tree".

**II - The appropriation of the HTPC system by professionals, between interests and mistrust**
II.A - A new social space serving the improvement of care and working conditions for professionals
II.A.1 - Inter-knowledge and coordination, knowing each other and working better together
Improve the quality of care
Improving your practice - HTPC seen as a tool for improving practices
Improve your working environment
Improve your working comfort
Facilitate social care
Do by mimicry, do like the others
Know each other better
Share knowledge and practices
Coordinate – work together
Reassure yourself about your practice
Coming together under social pressure
II.A.2 - The HTPC as validation of already existing work habits
HTPC primary care structuring action (consequence)
Comparisons of any kind with MSP
HTPC governance remains unclear
Working together generates daily conflict
Materialization or formalization of work habits
Primary care pre-structuring (prerequisite)
II.A.3 - Professional hierarchy as an obstacle to multi-professionalism
Defense of general medicine
Hierarchy between professions
Rare presence of specialists
Relationship between GP and specialists
II.A.4 - The HTPC, a new space for the defense of liberal medicine
Liberal medicine defense
Obstacles to coordination
Judicialization of the exercise
Attending physician measurement
Be stronger through regrouping
Fear of change
The economic engine of engagement
stowaway logic
A battle of age - generation effect
II.B - A territorial community conducive to the realization of projects
II.B.0 - Values of collective action
Conformity to values as a driver of engagement
Altruism as a driver of commitment
The desire to do as a driving force for commitment
Have a sense of collective
Political interest as a driver of commitment
II.B.1 - The project territory, when care meets health
Numerous public health actions
The example of prevention actions
Interest in city-hospital relations
Actions on the quality of care
Blood pressure self-measurement action
Actions around the health pathway
The missions of the HTPC
What vision of health

The transition from care to health, not trivial
II.B.2 - Access to care seen through the prism of medical demography rather than in terms of health inequalities
Background - medical demography
The HTPC favors new installations
Access to care is an issue for the HTPC
Priority access - actions towards migrants and precarious people - inequalities
Access to the GP gatekeeping mainly
Access to specialist doctors as working comfort for GPs
The limiting factor in medical demographics
Forgo home visits
Pragmatism as a driving force for commitment
Concern about territorial demography
II.B.3 - A plea for organizational flexibility, the HTPC as a model of adhocracy
Develop communication skills
The place of the leader not always clear
What participation of healthcare professionals within the system
II.C - The difficult projection of professionals at the territorial level
II.C.1 - A 'meeting in unknown territory'
The size of the HTPC raises questions
Fears towards other actors
Fear of other actors - elected officials
Fear other actors - hospital
Fear other actors - Order
*Fear of other actors – URPS**
ASV territories
*The preliminary or joint work of the CLS**
An obvious lack of knowledge of the HTPC system
Confusions over the objectives of the HTPC
Imperfect knowledge of the HTPC tool
Questions about the objective of the HTPC
II.C.2 - The discovery of the population dimension
Territorial dimension - population, to invest
Therapeutic education of the patient, on a national scale
Pooling the territory - shared spaces
The territory as a new horizon
II.C.3 - Fears of bureaucratization
Stacking devices
Fears of bureaucratization
Complexity in managing a large budget
Denunciation bureaucracy
"Reunionite" (= meeting addiction)
**III - Resources for successful institutionalization**
III.A - The request for autonomy as a condition of commitment
III.A.1 - The instituting power of partners
Power to establish partners
III.A.2 - Digital tools for healthcare professionals
Digital tools (DT)
DT - for the patient journey in the territory
DT - in the form of a social network
DT for coordination of actions
III.A.3 - Claiming know-how
Methodology skills
Project management skills

Know how to develop interventions
Field investigation
Interns' theses
The acquisition of specific know-how
Feedback
III.B - The need for coordination at the meso level
III.B.1 - Specific needs at the territorial level
Conflict management skills
Piloting skill
The need for coordination
The coordinator's profile
III.B.2 - An unsuitable coordination resource
EHESP (= French school of public health in Rennes) coordination training
III.B.3 - The coordination of HTPC, the affirmation of a new profession
New coordinator job
III.C - Limiting factors as obstacles to doing
III.C.1 - The difficult entry into the logic of contractualization
Contracting assistance skills
III.C.2 - The temporal resource, between impotence and delaying pretext
The time resource
III.C.3 - Access to data as a condition of empowerment
Access to data, essential
Activity data
ORS as a data popularization partner
Territorial project and diagnosis, using data to develop them
The example of unscheduled care and permanence of care
**IV - What support for professional dynamics in the HTPC**
IV.A - The strategic positioning of URPS*
IV.A.1 - A claimed deployment methodology
*Support from URPS**
IV.A.2 - The promotion of a liberal governance model
IV.A.3 - A vector of change in professional representations
IV.B - Support from regional health agencies
IV.B.1 - The regulator held remotely
*CPTS relations with the ARS**
IV.B.2 - The posture of "benevolent" support
*Support from the ARS**
Methodological support from the ORS (= regional observatory of health)
Use of consultants
IV.B.3 - Administrative constraint and injunction
Political pressure on administrations
IV.C - A misleading institutional wait-and-see attitude
IV.C.1 - The contribution of CPAM* to access to data
Health insurance support
IV.C.2 - Regional differences in terms of public action management
IV.C.3 - The importance of training in HTPC coordination
Competence in healthcare accounting and administration
Management skills
Evaluative skills
Conducting the evaluation
Data analysis
What training?

* The meanings of the abbreviations in English are provided in Appendix S2.1.
