# Supplementary material for: How Primary Healthcare Sector is Organized at the Territorial Level in France? A Typology of Territorial Structuring
Source: Int J Health Policy Manag. 2024 Jun 11;13:8231. doi: 10.34172/ijhpm.2024.8231 (PMC11270609; doi:10.34172/ijhpm.2024.8231)
Supplement: Supplementary file 3 — Recoding Variables. [file ijhpm-13-8231-s003.pdf]

Article title: How Primary Healthcare Sector is Organized at the Territorial Level in France? A Typology of Territorial Structuring

Journal name: International Journal of Health Policy and Management (IJHPM)

Authors' information: Sylvain Gautier[1,2]*, Loïc Josseran[1,2]

[1]Research Center in Epidemiology and Population Health, Primary Care and Prevention Team, Inserm U1018, Université Paris-Saclay, UVSQ, Villejuif, France.

[2]Department of Hospital Epidemiology and Public Health, Raymond Poincaré Hospital, GHU Université Paris-Saclay, AP-HP, Garches, France.

***Correspondence to:** Sylvain Gautier; Email: sylvain.gautier@uvsq.fr

Citation: Gautier S, Josseran L. How primary healthcare sector is organized at the territorial level in France? A typology of territorial structuring. Int J Health Policy Manag. 2024;13:8231. doi:10.34172/ijhpm.2024.8231

Supplementary file 3. Recoding Variables

Table S3.1: Recoding of the different quantitative variables used in the cluster analysis into qualitative variables with categories based on the values of their median and/or quartiles.

Table S3.1: Recoding of the different quantitative variables used in the cluster analysis into qualitative variables with categories based on the values of their median and/or quartiles.

The variable describing the density of pharmacies was presented with 4 categories: Low (< 27 pharmacies per 100,000 inhabitants), Medium ([27-33[), High ([33-42[), Very high (> 42). The variable describing the number of multi-professional structures was presented with 4 categories: "No multidisciplinary structure", "Only one", "Two", and "More than 2". The variable describing the evolution of the density of GPs between 2014 and 2019 was presented in 4 categories: Important decrease (negative variation < -14%), Decrease (negative variation > -14%), Stability (null variation), Increase (positive variation). The variable describing the evolution of the density of nurses was presented with 4 categories: Decrease (negative variation), Stability (null variation), Increase (< 36% of increase), Important increase (> 36% of increase). The variable describing the evolution of the density of midwives and physiotherapists was presented with 4 categories: Decrease or stability, Increase of midwives number, Increase of physiotherapist number, Increase of midwives and physiotherapist numbers. The variable describing the evolution of the density of pharmacies was presented with 3 categories: Decrease (negative variation), Stability (null variation), Increase (positive variation). The variable describing the evolution of the number and nature of multi-professional structures was presented with 6 categories: "No multidisciplinary structure", "Stability (no variation)", "New supply based on healthcare home", "Development of healthcare homes", "Development of healthcare centers", and "Development of healthcare homes and healthcare centers". The variable describing the evolution of the local health contract between 2014 and 2019 was presented with 4 categories: "No local health contract", "Old and not updated", "Old and updated", and "Recent (< 2 years) or recently updated (< 2 years)". The variable describing the longevity of multidisciplinary houses in the territory was presented with 4 categories: "No health house, "Opened more than 10 years ago", "Opened less than 10 years ago but more than 5 years", and "Opened within the last 5 years". The variable describing the longevity of health centers in the territory was presented with 4 categories: "No healthcare center", "Opened more than 20 years ago", "Opened less than 20 years ago but more than 5 years", and "Opened within the last 5 years". The variable describing the longevity of the HTPC in the territory was presented with 3 categories: "No HTPC", "Opened 2 or more years ago", and "Opened less than 2 years". Finally, the variable describing the longevity of the local health contract in the territory was presented with 4 categories: "No local health contract", "Opened 5 years ago or more", "Opened less than 5 years ago but more than 1 year ago", and "Opened 1 year ago or less".
