# Supplementary material for: How Primary Healthcare Sector is Organized at the Territorial Level in France? A Typology of Territorial Structuring
Source: Int J Health Policy Manag. 2024 Jun 11;13:8231. doi: 10.34172/ijhpm.2024.8231 (PMC11270609; doi:10.34172/ijhpm.2024.8231)

Article title: How Primary Healthcare Sector is Organized at the Territorial Level in France? A Typology of Territorial Structuring

Journal name: International Journal of Health Policy and Management (IJHPM)

Authors' information: Sylvain Gautier[1,2]*, Loïc Josseran[1,2]

[1]Research Center in Epidemiology and Population Health, Primary Care and Prevention Team, Inserm U1018, Université Paris-Saclay, UVSQ, Villejuif, France.

[2]Department of Hospital Epidemiology and Public Health, Raymond Poincaré Hospital, GHU Université Paris-Saclay, AP-HP, Garches, France.

***Correspondence to:** Sylvain Gautier; Email: sylvain.gautier@uvsq.fr

Citation: Gautier S, Josseran L. How primary healthcare sector is organized at the territorial level in France? A typology of territorial structuring. Int J Health Policy Manag. 2024;13:8231. doi:10.34172/ijhpm.2024.8231

Supplementary file 4. Multiple Correspondence Analysis and Hierarchical Clustering on Principal Components

Table S4.1: Correction of the explained variance for the MCA using Benzécri Correction and Greenacre Correction

Table S4.2: Correlations of the active variables with the first 5 factors (*i.e.* components of the MCA)

Table S4.3: Characteristics of the clusters of the territorial structuring of primary healthcare. Active variables used in the modelling. Mainland France, 2019

Figure S4.4: Scree plot.

Figure S4.5: Dendrogram.

Figure S4.6: Projection of the clusters onto the first factorial plane (factors 1 and 2)

Table S4.1: Correction of the explained variance for the MCA using Benzécri Correction* and Greenacre Correction**

Component	Benzécri Correction		Greenacre Correction	
	Explained variance	Cumulated explained variance	Explained variance	Cumulated explained variance
Dimension 1	30.0%	30.0%	19.0%	19.0%
Dimension 2	12.8%	42.8%	8.0%	27.0%
Dimension 3	8.1%	50.9%	5.2%	32.2%
Dimension 4	6.7%	57.6%	4.2%	36.4%
Dimension 5	5.4%	63.0%	3.4%	39.8%
Dimension 6	4.6%	67.6%	2.9%	42.7%
Dimension 7	3.9%	71.5%	2.4%	45.1%

*J. P. Benzecri, “Sur le calcul des taux d’inertie dans l’analyse d’un questionnaire”, *Cahiers de l’Analyse des Données*, 4(3) :377–378, 1979

** M.J. Greenacre, “Biplots in correspondence analysis”, *J. Appl. Stat.*, vol. 20, n°2, p. 251-269, Jan 1993

Table S4.2: Correlations of the active variables with the first 7 factors (*i.e.* components of the MCA)

	Factor 1 (8.7%)	Factor 2 (5.9%)	Factor 3 (4.9%)	Factor 4 (4.5%)	Factor 5 (4.1%)	Factor 6 (3.9%)	Factor 7 (3.6%)
**Primary healthcare services provision – healthcare professionals and services provision**							
Density of GPs	0,021	0,115	0,061	0,010	0,178	0,152	0,031
Density of healthcare professionals (HCP): GPs, midwives, physiotherapists, and nurses	0,240	0,213	0,125	0,005	0,062	0,038	0,005
Density of pharmacies	0,113	0,033	0,045	0,005	0,013	0,021	0,028
**Primary healthcare services provision – multidisciplinary structures provision**							
Number of multidisciplinary structures (healthcare homes, healthcare centers)	0,739	0,069	0,585	0,019	0,032	0,032	0,052
**Primary healthcare services provision – territorial context**							
Presence of a health territorial and professional community	0,037	0,000	0,001	0,872	0,001	0,009	0,059
Presence of a local health contract	0,317	0,554	0,085	0,008	0,005	0,002	0,000
**Primary healthcare services evolution (between 2014 and 2019)**							
Evolution of the density of GPs (%)	0,066	0,132	0,016	0,003	0,126	0,150	0,013
Evolution of the density of nurses (%)	0,037	0,014	0,011	0,003	0,014	0,017	0,016

	Factor 1 (8.7%)	Factor 2 (5.9%)	Factor 3 (4.9%)	Factor 4 (4.5%)	Factor 5 (4.1%)	Factor 6 (3.9%)	Factor 7 (3.6%)
Evolution of the density of midwives and physiotherapists (%)	0,080	0,096	0,027	0,005	0,086	0,051	0,013
Evolution of the density of pharmacies (%)	0,102	0,042	0,055	0,000	0,014	0,004	0,002
Evolution of the number and nature of multidisciplinary structures provision	0,670	0,115	0,636	0,030	0,642	0,196	0,419
Evolution of the local health contract(s)	0,335	0,577	0,098	0,081	0,090	0,445	0,271
**Primary healthcare services longevity**							
Longevity of healthcare center provision	0,423	0,126	0,019	0,008	0,205	0,084	0,110
Longevity of healthcare home provision	0,340	0,015	0,352	0,009	0,333	0,110	0,258
Longevity of health territorial and professional community provision	0,038	0,003	0,002	0,872	0,003	0,010	0,059
Longevity of local health contract	0,218	0,371	0,062	0,099	0,056	0,417	0,290

Table S4.3: Characteristics of the clusters of the territorial structuring of primary healthcare. Active variables used in the modelling. Mainland France, 2019

	Total N = 2,730 (100)	Cluster 1 n = 1,054 (38.6)	Cluster 2 n = 946 (34.7)	Cluster 3 n = 691 (25.3)	Cluster 4 n = 39 (1.4)
**Primary healthcare services provision – healthcare professionals and services provision**					
Density of GPs					
Low (< 63 GPs per 100,000 inhabitants	687 (25.2)	271 (25.7)	227 (24.0)	181 (26.2)	8 (20.5)
Medium ([63-102] GPs per 100,000 inhabitants)	1363 (49.9)	527 (50.0)	476 (50.3)	335 (48.5)	25 (64.1)
High (> 102 GPs per 100,000 inhabitants)	680 (24.9)	256 (24.3)	243 (25.7)	175 (25.3)	6 (15.4)
Density of healthcare professionals (HCP): GPs, midwives, physiotherapists, and nurses					
Low (≤ 34 HC Professionals per 100,000 inhabitants)	712 (26.1)	349 (33.1)	213 (22.5)	145 (21.0)	5 (12.8)
Medium (]34-55] HCPs per 100,000 inhabitants)	659 (24.1)	277 (26.3)	229 (24.2)	141 (20.4)	12 (30.8)
High (]55-90] HCPs per 100,000 inhabitants)	673 (24.7)	277 (26.3)	231 (24.4)	160 (23.2)	5 (12.8)
Very high (> 90 HCPs per 100,000 inhabitants)	686 (25.1)	151 (14.3)	273 (28.9)	245 (35.5)	17 (43.6)
Density of pharmacies					
Low (< 27 pharmacies per 100,000 inhabitants)	699 (25.6)	342 (32.4)	236 (24.9)	112 (16.2)	9 (23.1)
Medium ([27-33[pharmacies per 100,000 inhabitants)	629 (23.0)	265 (25.1)	212 (22.4)	144 (20.8)	8 (20.5)
High ([33-42[pharmacies per 100,000 inhabitants)	736 (27.0)	248 (23.5)	273 (28.9)	206 (29.8)	9 (23.1)
Very high (≥ 42 pharmacies per 100,000 inhabitants)	666 (24.4)	199 (18.9)	225 (23.8)	229 (33.1)	13 (33.3)
**Primary healthcare services provision – multidisciplinary structures provision**					
Number of multidisciplinary structures (healthcare homes, healthcare centers)					
No multidisciplinary structure	1062 (38.9)	1054 (100)	0 (0)	2 (0.3)	6 (15.4)
Only one multidisciplinary structure	841 (30.8)	0 (0)	533 (56.3)	298 (43.1)	10 (25.6)
Two multidisciplinary structures	403 (14.8)	0 (0)	234 (24.7)	162 (23.4)	7 (17.9)

	Total N = 2,730 (100)	Cluster 1 n = 1,054 (38.6)	Cluster 2 n = 946 (34.7)	Cluster 3 n = 691 (25.3)	Cluster 4 n = 39 (1.4)
More than 2 multidisciplinary structures	424 (15.5)	0 (0)	179 (18.9)	229 (33.1)	16 (41.0)
**Primary healthcare services provision – territorial context**					
Presence of a health territorial and professional community	39 (1.4)	0 (0)	0 (0)	0 (0)	39 (100)
Presence of a local health contract	987 (36.2)	275 (26.1)	0 (0)	691 (100)	21 (53.8)
**Primary healthcare services evolution (between 2014 and 2019)**					
Evolution of the density of GPs (%)					
Important decrease (negative variation < -14%)	712 (26.1)	266 (25.2)	230 (24.3)	203 (29.4)	13 (33.3)
Decrease (negative variation ≥ -14%)	598 (21.9)	169 (16.0)	238 (25.2)	181 (26.2)	10 (25.6)
Stability (null variation)	511 (18.7)	253 (24.0)	134 (14.2)	118 (17.1)	6 (15.4)
Increase (positive variation)	909 (33.3)	366 (34.7)	344 (36.4)	189 (27.4)	10 (25.6)
Evolution of the density of nurses (%)					
Decrease (negative variation)	347 (12.7)	164 (15.6)	102 (10.8)	79 (11.4)	2 (5.1)
Stability (null variation)	237 (8.7)	112 (10.6)	79 (8.4)	43 (6.2)	3 (7.7)
Increase (< 36% of increase)	1445 (52.9)	496 (47.1)	520 (55.0)	403 (58.3)	26 (66.7)
Important increase (≥ 36% of increase)	701 (25.7)	282 (26.8)	245 (25.9)	166 (24.0)	8 (20.5)
Evolution of the density of midwives and physiotherapists (%)					
Decrease or stability	588 (21.5)	265 (25.1)	187 (19.8)	129 (18.7)	7 (17.9)
Increase of midwives number	531 (19.5)	167 (15.8)	197 (20.8)	157 (22.7)	10 (25.6)
Increase of physiotherapist number	764 (28.0)	347 (32.9)	245 (25.9)	168 (24.3)	4 (10.3)
Increase of midwives and physiotherapist numbers	847 (31.0)	275 (26.1)	317 (33.5)	237 (34.3)	18 (46.2)
Evolution of the density of pharmacies (%)					
Decrease (negative variation)	436 (16.0)	122 (11.6)	164 (17.3)	142 (20.5)	8 (20.5)
Stability (null variation)	1746 (64.9)	754 (71.5)	590 (62.4)	382 (55.3)	20 (51.3)

	Total N = 2,730 (100)	Cluster 1 n = 1,054 (38.6)	Cluster 2 n = 946 (34.7)	Cluster 3 n = 691 (25.3)	Cluster 4 n = 39 (1.4)
Increase (positive variation)	548 (20.1)	178 (16.9)	192 (20.3)	167 (24.2)	11 (28.2)
Evolution of the number and nature of multidisciplinary structures provision					
No multidisciplinary structure	1062 (38.9)	1054 (100)	0 (0)	2 (0.3)	6 (15.4)
Stability (no variation)	613 (22.5)	0 (0)	361 (38.2)	243 (35.2)	9 (23.1)
New supply based on healthcare home	535 (19.6)	0 (0)	317 (33.5)	207 (30.0)	11 (28.2)
Development of healthcare homes	127 (4.7)	0 (0)	52 (5.5)	70 (10.1)	5 (12.8)
Development of healthcare centers	253 (9.3)	0 (0)	154 (16.3)	97 (14.0)	2 (5.1)
Development of healthcare homes and healthcare centers	140 (5.1)	0 (0)	62 (6.6)	72 (10.4)	6 (15.4)
Evolution of the local health contract(s)					
No local health contract	1743 (63.8)	779 (73.9)	946 (100)	0 (0)	18 (46.2)
Old and not updated	722 (26.4)	213 (20.2)	0 (0)	498 (72.1)	11 (28.2)
Old and updated	84 (3.1)	9 (0.9)	0 (0)	71 (10.3)	4 (10.3)
Recent (< 2 years) or recently updated (< 2 years)	181 (6.6)	53 (5.0)	0 (0)	122 (17.7)	6 (15.4)
**Primary healthcare services longevity**					
Longevity of healthcare home provision					
No "health house"	1571 (57.5)	1054 (100)	344 (36.4)	163 (23.6)	10 (25.6)
Opened more than 10 years ago	47 (1.7)	0 (0)	13 (1.4)	32 (4.6)	2 (5.1)
Opened less than 10 years ago but more than 5 years	456 (16.7)	0 (0)	220 (23.3)	225 (32.6)	11 (28.2)
Opened within the last 5 years	656 (24.0)	0 (0)	369 (39.0)	271 (39.2)	16 (41.0)
Longevity of healthcare center provision					
No healthcare center	1743 (63.8)	1054 (100)	391 (41.3)	281 (40.7)	17 (43.6)
Opened more than 20 years ago	620 (22.7)	0 (0)	330 (34.9)	277 (40.1)	13 (33.3)
Opened less than 20 years ago but more than 5 years	205 (7.5)	0 (0)	122 (12.9)	79 (11.4)	4 (10.3)

	Total N = 2,730 (100)	Cluster 1 n = 1,054 (38.6)	Cluster 2 n = 946 (34.7)	Cluster 3 n = 691 (25.3)	Cluster 4 n = 39 (1.4)
Opened within the last 5 years	162 (5.9)	0 (0)	103 (10.9)	54 (7.8)	5 (12.8)
Longevity of health territorial and professional community provision					
No HTPC	2691 (98.6)	1054 (100)	946 (100)	691 (100)	0 (0)
Opened 2 or more years ago	3 (0.1)	0 (0)	0 (0)	0 (0)	3 (7.7)
Opened less than 2 years	36 (1.3)	0 (0)	0 (0)	0 (0)	36 (92.3)
Longevity of local health contract					
No local health contract	1743 (63.8)	779 (73.9)	946 (100)	0 (0)	18 (46.2)
Opened 5 years ago or more	631 (23.1)	159 (15.1)	0 (0)	457 (66.1)	15 (38.5)
Opened less than 5 years ago but more than 1 year ago	345 (12.6)	112 (4.1)	0 (0)	228 (8.4)	5 (0.2)
Opened 1 year ago or less	11 (0.4)	4 (0.4)	0 (0)	6 (0.9)	1 (2.6)

Figure S4.4: Scree plot.

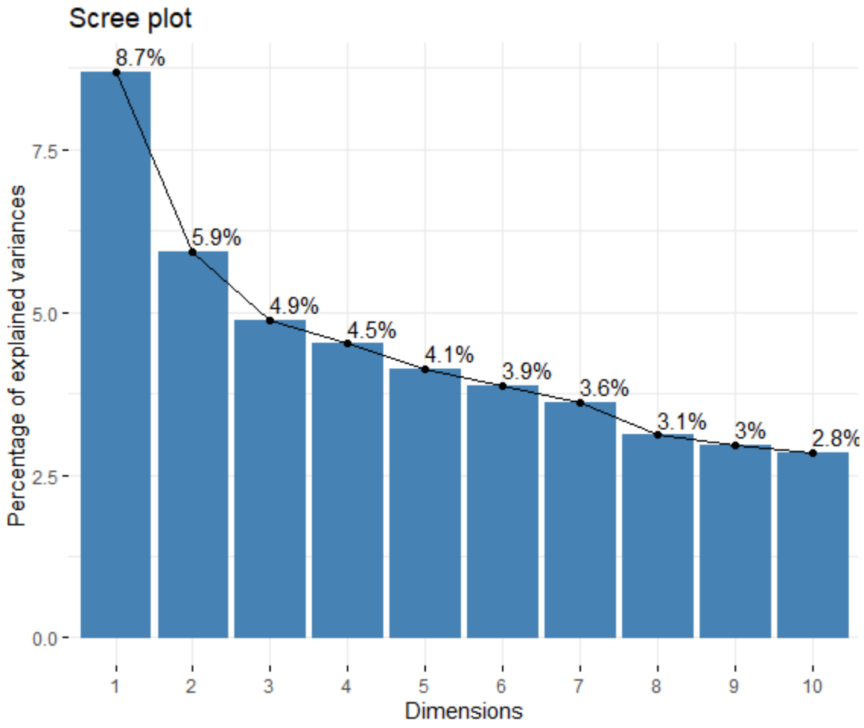

Figure S4.5: Dendrogram.

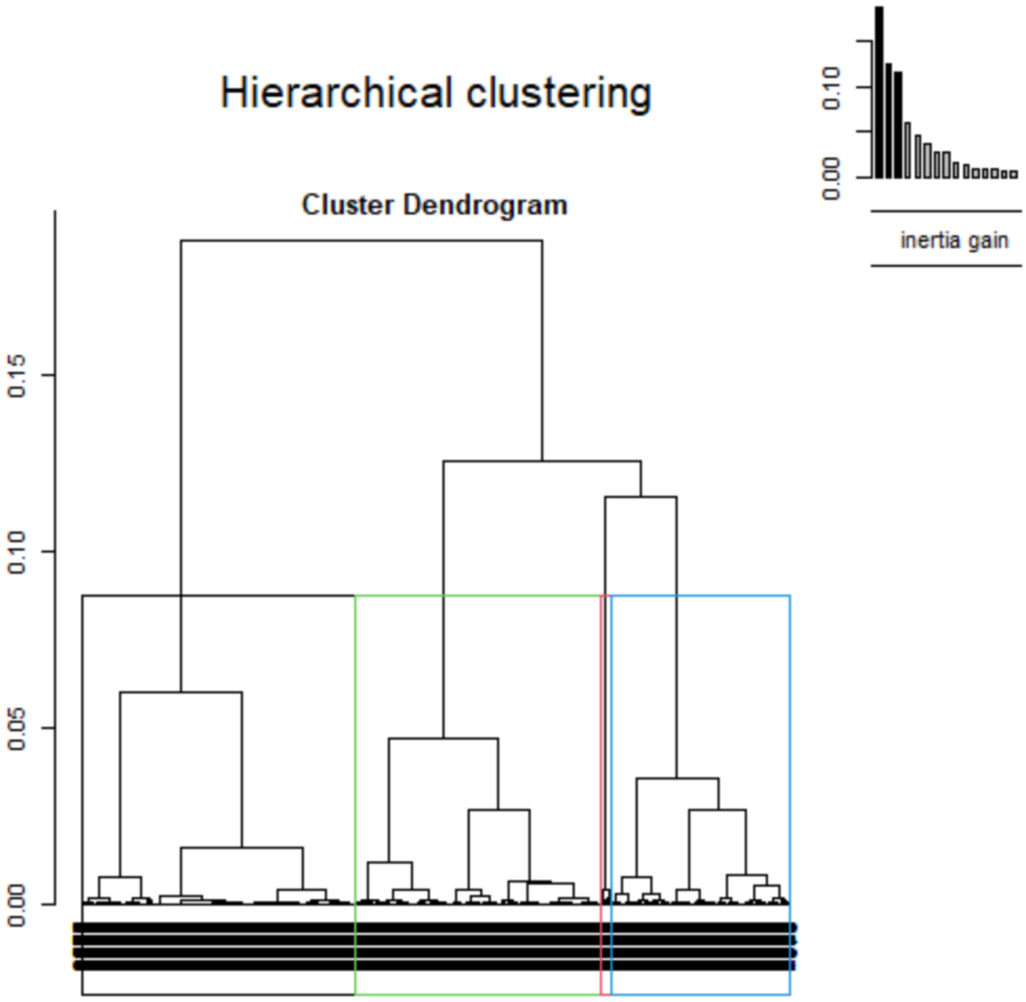

Figure S4.6: Projection of the clusters onto the first factorial plane (factors 1 and 2)

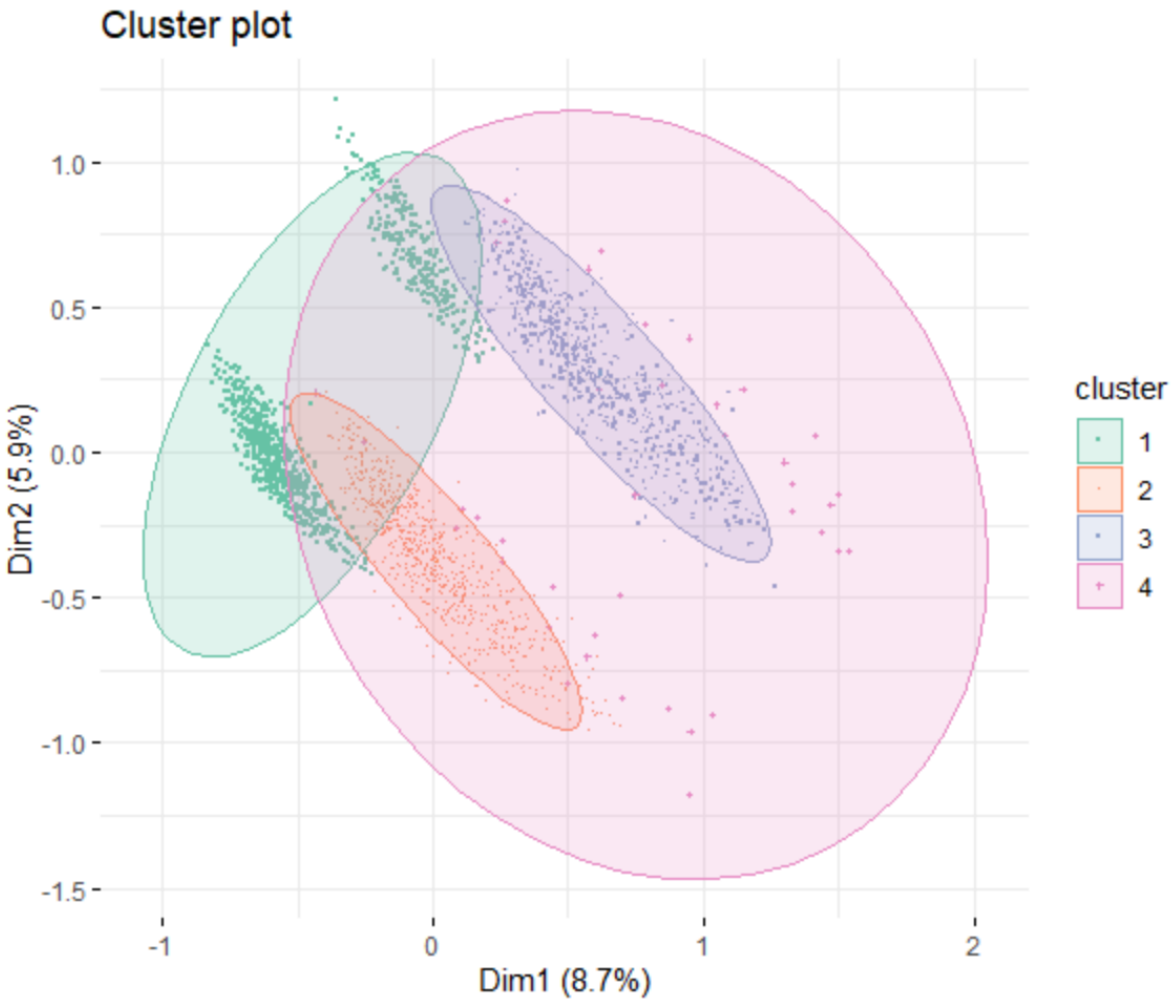

Supplement: Supplementary file 4 — Multiple Correspondence Analysis and Hierarchical Clustering on Principal Components. [file ijhpm-13-8231-s004.pdf]
